# Dr. Jules Guerin, on the Sanatory Condition of Paris during the First Six Months of 1842

**Published:** 1842-11-19

**Authors:** Jules Guerin


*On the Sanatory condition of Paris during the first six months of* 1842. By Dr. Jules Guerin.—In an interesting paper M. Guerin shows the relative mortality from all diseases during the first six months of 1842, as compared with those of 1841. The following table will best explain this. It enumerates the number of cases received, and the deaths which occurred in all the Parisian hospitals during the mentioned period.

| | 1841. | | 1842. | |
|---|---|---|---|---|
| Remaining on 31st Dec. | 5,430 | Deaths. | 5,160 | Deaths. |
| Admitted in January, | 6,290 | 593 | 6,550 | 570 |
| " February, | 5,772 | 539 | 6,099 | 566 |
| " March, | 6,689 | 632 | 6,630 | 673 |
| " April, | 6,503 | 664 | 6,859 | 706 |
| " May, | 6,931 | 659 | 6,877 | 674 |
| Total, | 37,615 | 3,087 | 38,175 | 3,189 |

This gives a general mortality of about one death in somewhat less than 12 cases for the first six months of 1841, and one death in a fraction more than 12 cases during the like period of 1842. It is stated that the general mortality during the four last months of both of these years was about one death in 16 cases. The deleterious action of atmospheric influences was more especially remarked on the very young and the aged. The following table, including the number of the patients, and the deaths which occurred in all the Parisian hospitals devoted to the cure of diseases of children, and of the aged, shows this. For the sake of comparison the tables are given for 1841 and 1842.

| | 1841. | | 1842. | |
|---|---|---|---|---|
| | No. of cases. | Deaths. | No. of cases. | Deaths. |
| Remaining 31st Dec. | 10,502 | | 10,070 | |
| Admitted January, | 869 | 331 | 974 | 332 |
| February, | 954 | 267 | 936 | 275 |
| March, | 1,090 | 266 | 1,099 | 309 |
| April, | 952 | 344 | 1,085 | 337 |
| May, | 967 | 199 | 980 | 382 |
| Total, | 15,334 | 1407 | 15,144 | 1635 |

Thus it appears, that, of the very young and the aged, there died in the ratio of 1 to 11 for 1841, and of 1 to 9 in 1842; and the meteorological tables which are appended show, that the last winter months and the first spring months of 1842 were more severe than those of 1841.

Puerperal fever seemed to follow the same general rule as other diseases, in so far as the mortality from it was concerned, as, both during the first six months of 1841 and of 1842, the chief mortality occurred during the month of April. The following table exhibits the number of deaths from puerperal fever, as compared with the number of deliveries in all the Parisian Lying-in-hospitals, during the first six months of 1841 and of 1842.

| | 1841. | | 1842. | |
|---|---|---|---|---|
| | No. of deliveries. | Deaths. | No. of deliveries. | Deaths. |
| January, | 310 | 13 | 329 | 7 |
| February, | 295 | 9 | 309 | 12 |
| March, | 327 | 15 | 335 | 32 |
| April, | 281 | 46 | 334 | 55 |
| May, | 318 | 7 | 286 | 35 |
| June, | 288 | 4 | 236 | 7 |
| Total, | 1819 | 94 | 1828 | 148 |

*Ibid, from Gazette Medicale de Paris,* August 6, 1842.

